# TEOA Inhibits Proliferation and Induces DNA Damage of Diffuse Large B-Cell Lymphoma Cells Through Activation of the ROS-Dependent p38 MAPK Signaling Pathway

**DOI:** 10.3389/fphar.2020.554736

**Published:** 2020-09-04

**Authors:** Xingxing Yu, Xin Wang, Xu Wang, Yi Zhou, Yanchun Li, Aiwei Wang, Tongtong Wang, Yihan An, Weidong Sun, Jing Du, Xiangmin Tong, Ying Wang

**Affiliations:** ^1^Clinical Research Institute, Zhejiang Provincial People’s Hospital, People’s Hospital of Hangzhou Medical College, Hangzhou, China; ^2^Department of Hematology, Fuyang Hospital of Anhui Medical University, Fuyang, China; ^3^School of Laboratory Medicine and Life Science, Wenzhou Medical University, Wenzhou, China; ^4^Department of Laboratory Medicine, Zhejiang Provincial People’s Hospital, People’s Hospital of Hangzhou Medical College, Hangzhou, China; ^5^Wangjiangshan Institute, Zhejiang Provincial People’s Hospital, People’s Hospital of Hangzhou Medical College, Hangzhou, China; ^6^The Second Clinical Medical School of Zhejiang Chinese Medical University, Zhejiang Chinese Medical University, Hangzhou, Zhejiang, China; ^7^Department of Hematology, The First People’s Hospital of Fuyang, Hangzhou, China; ^8^Phase I Clinical Research Center, Zhejiang Provincial People’s Hospital, People’s Hospital of Hangzhou Medical College, Hangzhou, China

**Keywords:** TEOA, diffuse large B-cell lymphoma, DNA damage, reactive oxygen species, p38 MAPK

## Abstract

Diffuse large B-cell lymphoma (DLBCL) is the most common subtype of lymphoma, accounting for approximately 30% to 40% of non-Hodgkin’s lymphomas (NHL). The administration of rituximab significantly improved the outcomes of DLBCL; however, the unavoidable development of resistance limits the long-term efficacy. Therefore, a new generation of less toxic drugs with higher chemotherapy response is required to prevent or reverse chemoresistance. TEOA is a pentacyclic triterpenoid compound isolated from the roots of *Actinidia eriantha*. Studies have confirmed that TEOA has significant cytotoxicity on gastrointestinal cancer cells. However, there are no relevant reports on DLBCL cells. In this study, we investigated the potential molecular mechanism of the anticancer activity of TEOA in DLBCL cells. The results demonstrated that TEOA inhibited proliferation and induced apoptosis in time-and dose-dependent manners. TEOA induced reactive oxygen species (ROS) generation, which was reversed by N-acetyl cysteine (NAC). TEOA induced DNA damage, increased the level of γ-H2AX, and the phosphorylation of CHK1 and CHK2. In addition, TEOA induced the activation of the p38 MAPK pathway and pretreated with p38 inhibitor SB20358 or ROS scavenger could block TEOA-induced DNA damage. Taken together, these results suggest that ROS mediated activation of the p38 MAPK signal pathway plays an important role in initiating TEOA-induced DNA damage.

## Introduction

DLBCL, the most common non-Hodgkin’s lymphoma in adults, with different clinical manifestations, histological features, and prognosis, is extremely harmful to human health (Jiang et al., 2017). In recent years, the incidence of DLBCL in China has been gradually increasing by more than 25,000 cases per year (Lenz and Staudt, 2010). The mortality rate of malignant lymphoma is 1.5/10 million people, ranking it 11 to 13 of all malignant tumors in China (Howlader et al., 2017). Currently, radiotherapy, chemotherapy, hematopoietic stem cell transplantation, targeted therapy, and immunotherapy are the primary treatment strategies for DLBCL (Wang et al., 2018). However, targeted drugs are only effective against specific types of lymphoma, and hematopoietic stem cell transplantation is limited by donors. Therefore, chemotherapy is still the most commonly used primary treatment method of most types of lymphoma. Some patients with diffuse large B-cell lymphoma can achieve complete remission through chemotherapy in a short period (Juul et al., 2018). Due to the severe toxic side effects of chemotherapy drugs, and high relapse rates, most patients are eventually forced to discontinue treatment, and the long-term survival rate is only 30% to 50% (Chihara et al., 2016). Thus, the search for new effective and low-toxic anticancer drugs is a critical topic in the field of DLBCL research.

Herbal and natural products have been demonstrated to be a valuable source for anticancer drug screening in recent years (Mondal et al., 2012). TEOA (2α, 3α, 24-thrihydroxyurs-12-en-24-ursolic acid), a pentacyclic triterpenoid isolated from the roots of *Actinidia eriantha*, exhibited significant biological activity against various diseases, including hepatitis, edema, rheumatoid arthritis, dysentery, and lymphoid tuberculosis (Liang et al., 2007). Recent studies indicated that TEOA potentially possesses anticancer activity (Zhou et al., 2010). Furthermore, TEOA-induced cell death is mediated by the generation of reactive oxygen species (ROS) in gastrointestinal cancer (Zhang et al., 2017). However, its anticancer effect in DLBCL remains unknown.

Genomic instability is considered an important feature of cancer, which demonstrates a trend in the accumulation of DNA damage with the occurrence of mutations, genomic abnormalities, and metastatic phenotypes, ultimately favoring development of cancer and drug resistance (Halazonetis et al., 2008; Negrini et al., 2010). DLBCL is a heterogeneous disease characterized by high levels of genomic instability, and the activation of DNA damage repair pathways (Jong et al., 2020). ROS is the main molecule produced in the body during oxidative stress, and it plays an essential role in various life processes of cells (Scherzshouval and Elazar, 2011). It can serve as a second messenger to regulate signal transduction, related to cell proliferation, differentiation, apoptosis, and autophagy (Djavaheri-Mergny et al., 2007; Liu et al., 2009). Numerous studies have shown that ROS can attack the DNA, causing DNA damage, such as DNA strand breaks, DNA site mutations, DNA double-stranded aberrations, proto-oncogenes, and tumor suppressor gene mutations (Ren et al., 2016). The MAPK family is a group of serine/threonine kinases, including the extracellular signal-regulated kinase pathway, c-Jun N-terminal kinase pathway, and p38 MAPK pathway. The p38 MAPK pathway plays a vital role in apoptosis, cytokine production, transcription regulation, and cytoskeletal reconstruction (Suzuki et al., 2001). Activation of p38 MAPK can augment the processes of apoptosis. Meanwhile, MAPK signal transduction cascades can be regulated by ROS (Xie et al., 2016). However, the relationship between the activation of p38 MAPK and DNA damage remains elusive. In this study, we investigated the molecular mechanism underlying TEOA-induced apoptosis through the ROS/p38 pathway and DNA damage in DLBCL. Currently, tumor resistance is a major clinical challenge (Jing et al., 2016). Interestingly, we found that TEOA has a synergistic effect with vindesine and cyclophosphamide; the combination therapy could increase the synthetic lethal effect on DLBCL cells.

## Materials and Methods

### Cell Culture

OCI-LY3 and OCI-LY10 cells were obtained from the Cell Bank of Type Culture Collection of Chinese Academy of Sciences (Shanghai, China). Cells were cultured in RPMI-1640 medium (Hyclone, USA) with, 10% fetal bovine serum (Gibco, USA), 1% penicillin/streptomycin (Beyotime, Shanghai), and 0.5% Glutamine (Beyotime, Shanghai) in 5% CO2 at 37°C. All cells were used within 20 passages.

### Reagents and Antibodies

OCI-LY3 and OCI-LY10 cells were kindly provided by professor Wenbin Qian of Zhejiang University (Zhejiang, China). Z-VAD-FMK and SB203580 were obtained from Selleck Chemicals (Houston, TX). CCK-8 Assay kit was purchased from Meilunbio (Dalian, China). N-acetylcysteine (NAC) and glutathione (GSH) were obtained from Abcam (Cambridge, MA). The Cell Apoptosis detection kit and Cell Cycle Staining Kit were purchased from MultiSciences (Hangzhou, China). Primary antibodies, including anti-cleaved-PARP (ab191217), anti-cleaved-Caspase 3(ab32042), anti-Bax (ab32503), anti-Bad(ab32445), anti-Bcl2(ab32124), anti-β-Actin(ab179467), anti-p21(ab109520), anti-p27(ab32034), anti-p53 (ab179477), anti-p38(ab170099), anti-chk1(ab40866), anti-p-chk2, and anti-γ-H2AX, were purchased from abcam (Cambridge, MA). Antibodies including anti-p-p38(9215S), anti-chk2(3440S), anti-p-chk1(2348P) were purchased from Cell Signaling Technology (Massachusetts, USA). Secondary antibodies, including goat anti-rabbit and goat anti-mouse IgG-HRP were obtained from Beyotome (Shanghai, China).

### Cell Viability Assay

Cell viability was detected by Cell Counting Kit 8. OCI-LY3 and OCI-LY10 cells were seeded onto 96-well plates at a density of 4 × 10^4^ cells/well with 100 µl Serum-free medium, and treated with increasing concentrations of TEOA (5, 10, 15, 20, 25, 30, 35, 40, 45, and 50 µM) for indicated time. Then, 10 µl cell counting kit-8 was added to each well and incubated for 2 h at 37°C. The absorbance was measured at 450 nm using the microplate reader.

### Cell Apoptosis Assay

Cell apoptosis was determined by the Annexin V-FITC/PI apoptosis kit. The OCI-LY3 and OCI-LY10 cells were cultured in 6-well plates at a density of 1.5 × 10^6^ cells/well, and treated with the indicated concentrations of TEOA for 12 h. Then, the cells were collected, washed once with PBS, and mixed with 500 µl of binding buffer. After incubating with 5 µl Annexin V-FITC and 10 µl PI for 5 min at room temperature in the dark, the cell apoptosis was assessed by flow cytometry (ACEA NovoCyte, USA).

### Soft Agar Colony Assay

A soft agar colony assay was used to detect the long-term effects of TEOA in OCI-LY3 and OCI-LY10 cells. Cells were seeded into 6-well plates at 2,000 cells/well, with 0.48% top agar layer and 1% bottom agar layer (Agarose, Sigma-Aldrich), and treated with different concentrations of TEOA in RPMI-1640 medium with 10% fetal bovine serum (FBS). The cell colonies were formed following incubation for 2 weeks. Thereafter, they were fixed with 4% paraformaldehyde (Sigma-Aldrich, USA) for 15 min, and stained with crystal violet (Beyotime, China) for 15 min at room temperature.

### Western Blotting

Cells were treated with the indicated concentrations of TEOA for 12 h. Then, the cells were collected and washed once with ice-cold PBS and lysed in RIPA lysis buffer (Beyotime, China) for 10 min on ice. The mixture was centrifuged at 14,000 rpm/min, and 4°C (Thermo Fisher, USA) for 10 min. The supernatant was removed, and the cellular protein concentration was measured using the bicinchoninic acid protein assay kit (Thermo Fisher, USA). For western blot analysis, 10 µg protein was loaded into each lane and separated using sodium dodecyl sulfate-polyacrylamide gel electrophoresis (SDS-PAGE) and then transferred onto Polyvinylidene fluoride (PVDF) membranes (Bio-Rad, USA). After blocking with 5% non-fat milk at room temperature for 1 h, the membranes were incubated overnight with the primary antibodies at 4°C. They were subsequently washed with TBST three times, and the membranes were incubated with secondary antibodies at room temperature for 1 h. The expression of target bands was detected using an enhanced chemiluminescence system (Bio-Rad, USA). The working concentration of primary antibodies was 1:1,000, except where illustrated. The primary antibodies used in this experiment: anti-cleaved-PARP, anti-Bax, anti-Bad, anti-cleaved-Caspase 3, anti-Bcl2, anti-p21, anti-p27, anti-p53, anti-p-p38, anti-p38, anti-p-chk1, anti-chk1, anti-p-chk2, anti-chk2, anti-γ-H2AX(1:2,000), and anti-β-Actin(1:6,000). The secondary antibodies were goat anti-rabbit IgG (H+L) (1:2,000) and goat anti-mouse IgG (H+L) (1:2,000). β-actin served as the loading control. Statistical analysis was performed using Image J software.

### Reactive Oxygen Species (ROS) Analysis

The intracellular ROS level was detected using an oxidation sensitive fluorescent probe (DCF-DA) (Beyotime, China) according to the manufacturer’s instructions. DCF-DA can be hydrolyzed by intracellular esterase to form DCFH; it can be oxidized by reactive oxygen to produce the fluorescent compound, 2′,7′-dichlorofluorescein (DCF), which is a stable fluorescent ROS-sensitive compound that can readily permeate into cells. OCI-LY10 cells were incubated with the indicated concentrations of TEOA in serum-free medium for 12 h. Then, cells were harvested and washed thrice with serum-free medium. Thereafter, cells were resuspended in 1640 medium and stained with DCF-DA (4 µM) for 30 min at 37°C. Lastly, cells were washed thrice with serum-free medium and fluorescence intensity was measured by flow cytometry.

### Propidium Iodide (PI) Staining

The cells were seeded into 96-well plates at a density of 4 × 10^4^ cells/well, and treated with the indicated concentrations of TEOA for 12 h. Then, PI was added to each well at a final concentration of 10 µg/ml and incubated at 37°C for 10 min in the dark. Images were obtained using a fluorescence microscope (Nikon, Germany).

### Hoechst 33258 Staining

The cells were seeded into 24-well plates at a density of 2 × 10^5^ to 3 × 10^5^ cells/well and treated with the indicated concentrations of TEOA for 12 h. Cells were collected and stained with Hoechst 33258 for 5 min, then washed thrice with PBS. The blue nuclei fluorescence was detected by confocal laser scanning microscopy (Leica, Germany).

### Immunocytochemistry

The formation of γ-H2AX was detected by immunocytochemistry. The cell slides were treated with polylysine for 24 h. Simultaneously, OCI-LY10 cells were seeded into 6-well plates and treated with the indicated concentrations of TEOA for 12 h. Then cells were harvested and seeded on cell slides and incubated for 2 h. Cells were fixed with 4% paraformaldehyde for 15 min and permeabilized with 1% Triton X-100 for 5 min at room temperature. After being blocked with 5% BSA, the cells were stained with 1:200 of γ-H2AX primary antibody and 1:500 of FITC-labeled Goat Anti-Rabbit IgG (H+L) secondary antibody (Beyotime, USA). DAPI was used for the staining of the nucleus. Images were acquired using confocal microscopy (Leica, Germany).

### Cell Cycle Analysis

After indicated treatment for12 h, cells were harvested and fixed in ice-cold 70% ethanol overnight at 4°C. Next day, the ethanol was removed by centrifugation, cells were washed with PBS and re-suspended in PBS containing PI (50 mg/ml) and RNase A (10 mg/ml) and then incubated at 37°C for 30 min in the dark. After washing and filtration, the single-cell suspensions were subjected to the flow cytometry (ACEA NovoCyte, USA) and the percentage of cells at G0/G1, S, or G2/M phase were quantified.

### Combination Index of TEOA With Chemotherapeutic Drugs

The therapeutic effect of TEOA in combination with vindesine or cyclophosphamide was measured by the CCK-8 assay. 15 µM TEOA treated with increasing concentrations of CTX(0.5, 1, 2 µM)or Vindesine(16, 24, 32 µg/ml)for 12 h and combination index was calculated through the formula: Q= E(A+B)/(E A +E B -E A ×E B). EA and EB represent the inhibition ratio of drug A and drug B on cells respectively, and E(A+B) is the combined inhibition effect of drug A and B. Q>1.15, = 0.85~1.15 and <0.85 indicate synergistic effect, additive effect and antagonistic effect, respectively.

### Data Analysis

The effects of various *in vitro* drug treatments were compared by Student’s *t*-test using GraphPrism.5. All results were presented as the mean  ±  standard deviation (SD). Differences with *p* < 0.05 were considered statistically significant.

## Results

### TEOA Inhibited the Cell Viability of DLBCL Cells

**Figure 1:**
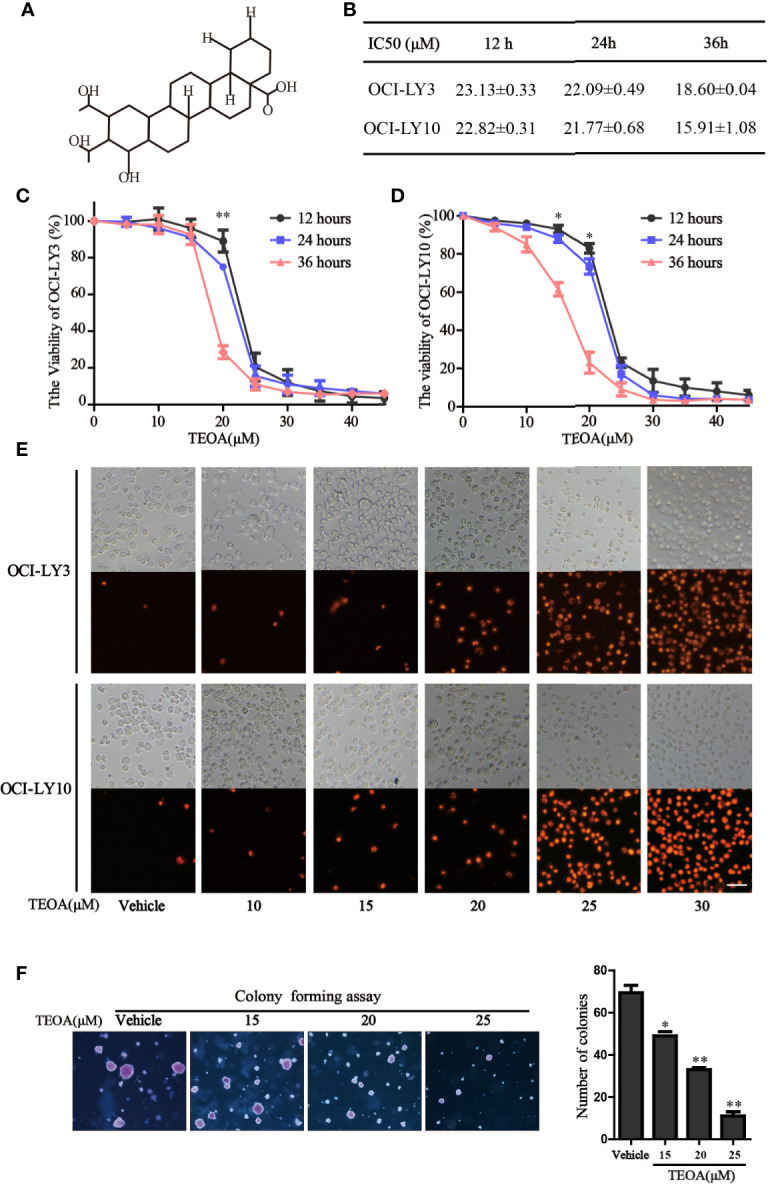
TEOA reduced diffuse large B-cell lymphoma (DLBCL) cell viability. **(A)** The chemical structure of TEOA. **(B)** The calculated IC50s of TEOA at 12 h, 24 h, and 36 h in OCI-LY3 and OCI-LY10 cells. **(C, D)** OCI-LY3 and OCI-LY10 cells were treated with TEOA at various concentrations (0, 5, 10, 15, 20, 25, 30, 35, 40, and 45 μM) for 12 h, 24 h, and 36 h; cell viability was detected by CCK8 assays. **(E)** The OCI-LY3 and OCI-LY10 cells were treated with indicated concentrations of TEOA for 12 h, then stained with propidium iodide (PI) and photographed under fluorescence microscopy; scale bar: 40µm. **(F)** The colony formation of OCI-LY10 cells treated with indicated concentrations of TEOA for 14 days. The colonies were photographed by microscope; the corresponding statistical graph was showed on the right. Data were presented as mean ± SD of three independent experiments, *P<0.05, **P<0.01.

The chemical structure of TEOA is indicated in **Figure 1A**. To examine the inhibitory effect of TEOA on cell viability, OCI-LY3 and OCI-LY10 cells were seeded in 96-well plates and treated with increasing concentrations of TEOA (0, 5, 15, 20, 25, 30, 35, 40, 45, or 50 µM) for indicated times. Cell viability was measured by the CCK-8 assay. Results showed that TEOA significantly inhibited cell viability in a dose and time-dependent manner (**Figures 1C, D**). The half-maximal inhibitory concentration (IC50) of TEOA at 12 h, 24 h, and 36 h were calculated and shown in **Figure 1B**. Further, we observed morphological changes by phase-contrast microscopy and found the cells were shattered, metamorphous and multidirectional after TEOA treatment. Moreover, the number of PI-positive cells was increased in a dose-dependent manner (**Figure 1E**). The soft agar clone formation assay was performed to determine the long-term growth inhibitory effect of TEOA. The OCI-LY10 cells were treated with increasing concentrations of TEOA (0, 15, 20, and 25 µM) in 0.48% agarose with 10% FBS for 14 days; the results revealed that TEOA significantly inhibited clone formation (**Figure 1F**). The clones were counted and corresponded quantification histograms were shown on the right. In addition, the effect of TEOA on non-cancerous cell lines was also detected and the results shown that TEOA exhibited lower toxicity on mouse embryonic fibroblast and immortalized lymphocyte cells (**Figure S1A**). To determine whether TEOA decreased cell viability by affecting the cell cycle distribution or not. The cell cycle distribution was performed and revealed that cells were arrested at G0/G1 phase and the proportion was increased in a dose-dependent manner (**Figures S1D**). In addition, TEOA inhibited cell migration rate by approximately 30% and 40% at the doses of 20 and 25μM, respectively (**Figure S1E**). Taken together, these results suggest that TEOA reduced the viability and inhibited cell proliferation of DLBCL cells.

### TEOA Activated Apoptosis of DLBCL Cells

**Figure 2:**
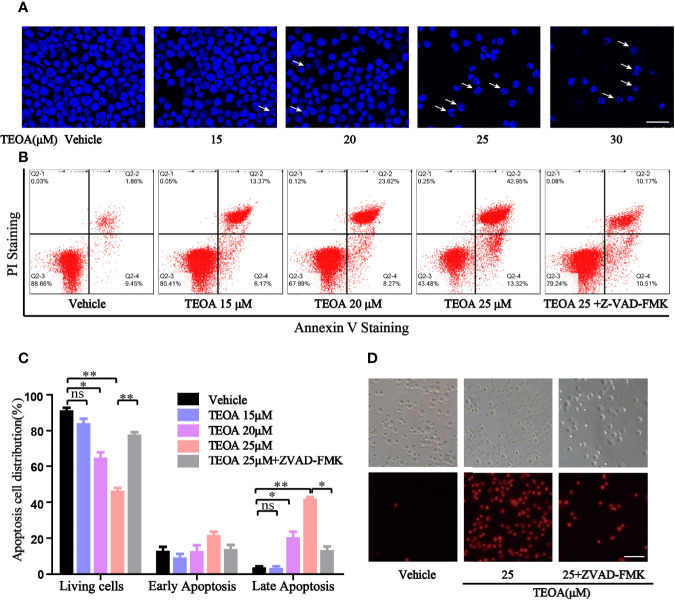
TEOA induced apoptosis in diffuse large B-cell lymphoma (DLBCL) cells. **(A)** OCI-LY10 cells treated with indicated concentration of TEOA for 12 h were stained with Hoechst 33258, and the nuclear fragmentation was indicated with white arrows; scale bar: 40 µm. **(B, C)** OCI-LY10 cells were pretreated with or without z-VAD-FMK (5 μM) for 1 h, then treated with different concentrations of TEOA for 12 h; the results were analyzed by flow cytometry with Annexin V-FITC/PI apoptosis kit. The proportion of apoptotic cells was presented by GraphPad Prism5; *P<0.05, **P<0.01. **(D)** OCI-LY10 cells were treated with 25μM TEOA in the presence or absence of 5μM z-VAD-FMK for 12 h, cell death was detected by PI staining; scale bar: 40 µm.

**Figure 3:**
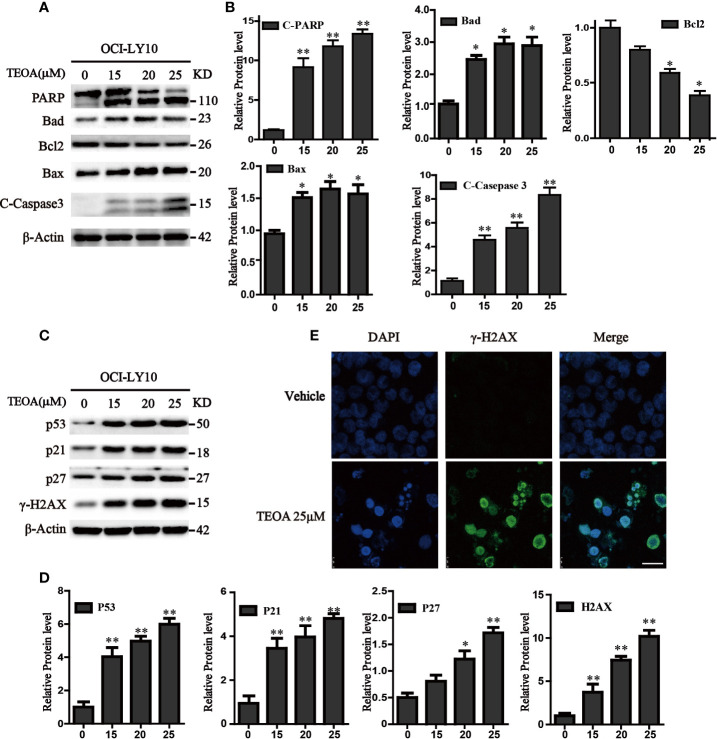
TEOA affected the expression of apoptosis-related protein in OCI-LY10 cells. **(A)** Diffuse large B-cell lymphoma (DLBCL) cells were exposed to various concentrations of TEOA for 12 h. The expression of the following apoptosis-related proteins was determined by western blot: cleaved PARP, caspase-3, and Bcl-2 family members Bcl-2, Bad, and Bax; β-actin was used as a loading control. **(B)** The quantitation of cleaved PARP, caspase-3, Bcl-2, Bad, and Bax were shown. *P<0.05 **P<0.01. **(C)** OCI-LY10 cells were treated with different doses of TEOA for 12 h. The expression of P53, P21, P27, and γ-H2AX protein was determined by western blot; β-actin was used as a loading control. **(D)** The quantitation of P53, P21, P27, and γ-H2AX. Data were presented as mean ± SD. *P<0.05, **P<0.01. **(E)** OCI-LY10 cells were treated with 25 µM TEOA for 12 h and stained with γ-H2AX (1:200) primary antibody and FITC-labeled Goat Anti-Rabbit IgG (H+L) (1:500) antibody, DAPI was used for nucleus staining. Images were acquired with the confocal laser scanning microscopy; scale bar: 10 µm.

To further determine whether TEOA decreased cell viability through the regulation of apoptosis. OCI-LY10 cells were treated with TEOA at concentrations of 0, 15, 20, 25, and 30 μM, respectively, and followed by apoptosis detection and Hoechst 33258 staining. The results showed that, after treatment with TEOA overnight, the percentage of apoptotic cells significantly increased in a concentration-dependent manner, accompanied by nuclear fragmentation and condensation (**Figure 2A**, white arrow). As shown in **Figures 2B, C**, we observed that TEOA induced significant apoptosis in DLBCL cells, detected by Annexin-V and PI staining followed by flow cytometry assays. Furthermore, the apoptotic rate significantly increased with the increasing concentration of TEOA. Meanwhile, to determine whether apoptosis induced by TEOA was caspase-dependent, the cells were pretreated with the pan-caspase inhibitor, ZVAD-FMK, (5 μM) for 1 h. Importantly, we found that the rate of apoptosis was significantly decreased by ZVAD-FMK pretreatment, especially in late apoptosis (**Figure 2C**). Moreover, the results of PI staining also demonstrated that ZVAD-FMK rescued TEOA-induced apoptosis (**Figure 2D**). These findings indicate that TEOA-induced apoptosis in DLBCL is dependent on the caspase pathway. Next, we investigated the expression of apoptosis-associated proteins by western blot. The results showed TEOA treatment induced the cleavage of caspase-3 and PARP, up-regulated the expression of Bad, and Bax, and down-regulated the expression of Bcl2 (**Figures 3A, B**). Taken together, our findings suggest that TEOA promotes apoptosis in DLBCL cells.

### TEOA Induced DNA Damage in DLBCL Cells

Accumulating evidence indicates that apoptosis is mediated by some pro-apoptotic genes, such as p53. Therefore, we detected the expression of p53 and found that TEOA upregulated the level of p53 protein. Simultaneously, we observed that the expression of P21, P27, and γ-H2AX was also increased (**Figures 3C, D**). As a transcription factor, p53 could not only active apoptosis but also induces cell cycle arrest in response to DNA damage. γ-H2AX is a product of DNA fragmentation, which can be detected in the early stages of DNA damage. Additionally, we observed that TEOA increased the co-staining of DAPI and γ-H2AX foci in OCI-LY10 cells (**Figure 3E**). And the level of 8-hydroxy-2’-deoxyguanosine (8-OHdG), which is a modified nucleoside base and one of the most commonly studied byproduct of DNA damage, was also elevated under the treatment of TEOA (**Figure S2**). Thus, these results suggest that TEOA could induce DNA damage in DLBCL cells.

### TEOA Induced Intracellular ROS and Promoted Apoptosis in OCI-LY10 Cells

**Figure 4:**
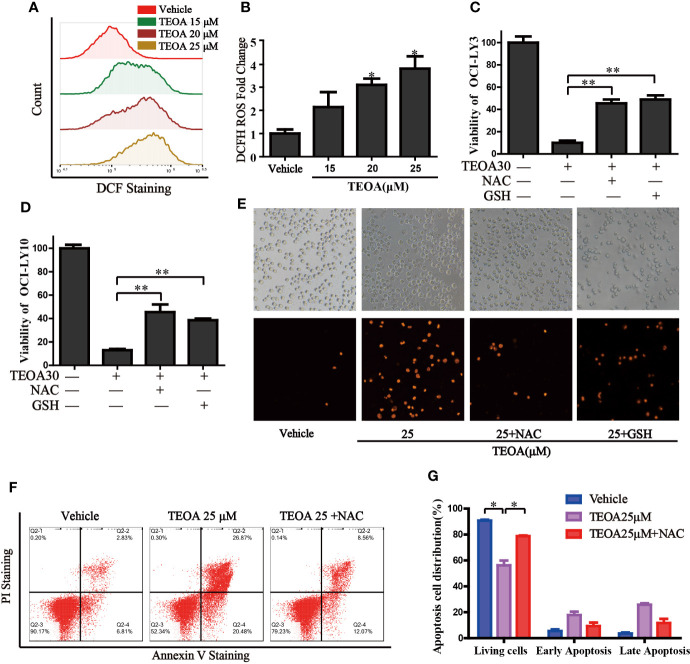
TEOA increased the production of cellular ROS and promoted apoptosis. **(A)** OCI-LY10 cells were exposed to different concentrations of TEOA for 12 h, then treated cells were stained with DCF-DA for 30 min, cellular ROS levels were determined by flow cytometry. **(B)** Quantitative analysis of the cellular ROS, *P<0.05. **(C, D)** OCI-LY10 and OCI-LY3 cells were treated with TEOA for 12 h in the presence or absence of NAC and GSH, cell viability was determined by CCK8 assay and PI staining **(E)**. **(F)** Flow cytometry was used to detect apoptosis in DLBCL cells exposed to TEOA with or without NAC treatment. **(G)** The proportion of apoptotic cells was shown on the right, *P<0.05, **P<0.01.

Next, we investigated intracellular signaling events upon TEOA-induced DNA damage and apoptosis. We detected intracellular ROS levels using a DCF-DA probe. ROS was significantly activated by the treatment of TEOA (**Figures 4A, B**). The use of ROS scavenger, NAC, and GSH could markedly decrease TEOA-induced cell death, as shown by the CCK-8 assay (**Figures 4C, D**), demonstrating that the intracellular signaling events induced by TEOA were ROS dependent. To further confirm whether increased ROS promotes apoptosis, we detected apoptosis by flow cytometry (**Figures 4F, G**). Compared with the control group, the apoptosis rate with TEOA was significantly increased, while adding ROS scavenger NAC significantly inhibited apoptosis of OCI-LY10 cells, suggesting that TEOA-induced apoptosis of OCI-LY10 cells was related to ROS. And this phenomenon was further confirmed by PI staining (**Figure 4E**). These data indicate that the TEOA-induced ROS accumulation might be a potential mechanism underlying the anti-DLBCL activity.

### TEOA Induced DNA Damage Through p38 MAPK Activation and Upregulated the Phosphorylation of p-CHK1 and p-CHK2 in DLBCL Cells

**Figure 5:**
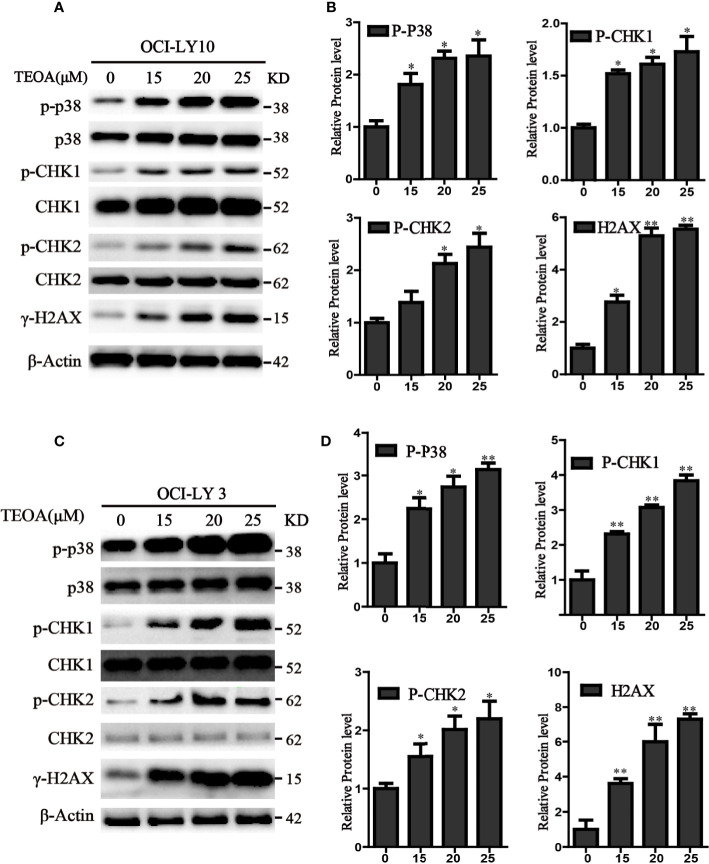
TEOA induced DNA damage in diffuse large B-cell lymphoma (DLBCL) cells. **(A, C)** OCI-LY10 and OCI-LY3 cells were treated with TEOA for 12 h and then subjected to western blot with staining for p-p38, p38, p-CHK1, CHK1, p-CHK2, CHK2, and γ-H2AX. **(B, D)** Analysis of the expression of p-p38, p-CHK1, p-CHK2, and γ-H2AX by Image J software. Data were presented as mean ± SD, *P<0.05, **P<0.01.

**Figure 6:**
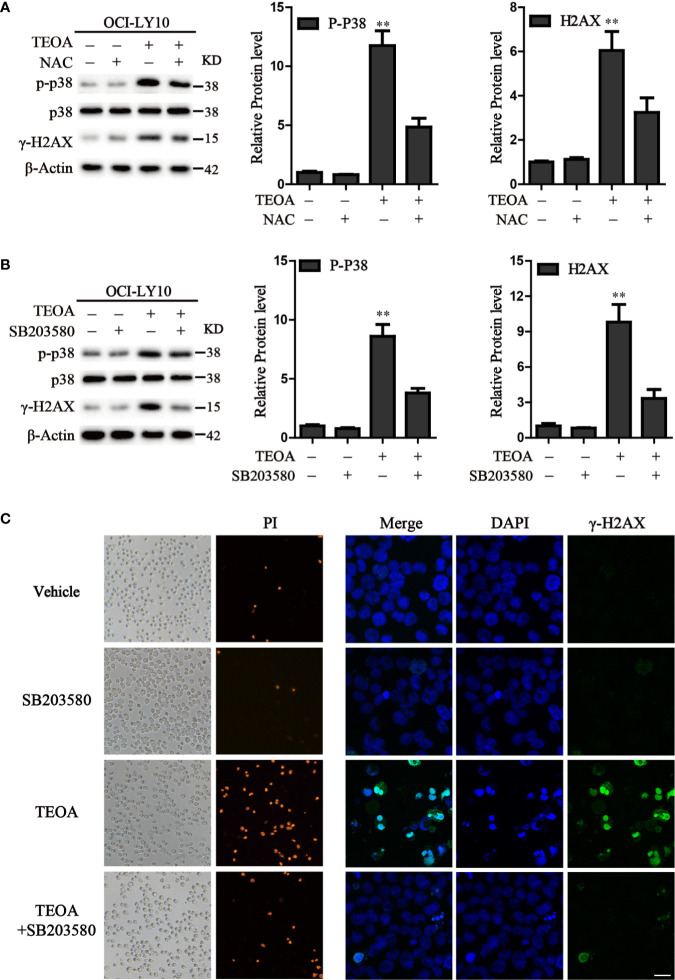
Inhibition of ROS accumulation and p38 activation suppressed apoptosis induced by TEOA. **(A)** OCI-LY10 cells were exposed to TEOA for 12 h with or without NAC (5 mM) pretreatment for 1 h, the levels of p-p38, total p38, and γ-H2AX were determined by western blot. The expression of p-p38 and γ-H2AX was quantified by Image J software. Data were presented as mean ± SD, **P<0.01. **(B)** OCI-LY10 cells were exposed to TEOA for 12 h with or without SB203580 (10 μM) incubation in advance for 1 h, the levels of p-p38, total p38, and γ-H2AX were determined by western blot. Corresponding expression analysis of p-p38 and γ-H2AX were shown on the right. Data are presented as mean ± SD, **P<0.01. **(C)** OCI-LY10 cells were treated with 25 µM TEOA for 12 h with or without SB203580 (10 μM) pretreatment for 1 h and stained with PI or γ-H2AX (1:200) primary antibody and FITC-labeled Goat Anti-Rabbit IgG (H+L) (1:500) secondary antibody. DAPI was used for nucleus staining. Images were acquired with a confocal laser scanning microscopy; scale bar: 10µm.

P38 kinase is a member of the mitogen-activated protein kinase (MAPK) family, and plays a pivotal role in apoptosis (Yuan et al., 2016). In addition to DNA damage, we analyzed the changes in the MAPK pathway after treatment with TEOA, and examined the relationship between p38 MAPK activation and DNA damage induced by TEOA. Interestingly, we found that TEOA could activate the p38 MAPK pathway by increasing the phosphorylation of p38, CHK1, and CHK2, and improve the expression of γ-H2AX in a dose-dependent manner in OCI-LY10 (**Figures 5A, B**) and OCI-LY3 cells (**Figures 5C, D**). These results indicate that TEOA activates the p38 MAPK pathway and induces DNA damage repair. Furthermore, the active oxygen scavenger, NAC, significantly downregulated the expression of p-p38 and decreased DNA damage (**Figure 6A**). The p38 phosphorylation inhibitor SB203580 could block TEOA-induced DNA damage and reduce the expression of p-p38 (**Figure 6B**). Further, immunofluorescence was used to detect the effect of SB203580 on reversing DNA damage. Results showed that TEOA-induced γ-H2AX accumulation were indeed blocked in the treated cells with p38 inhibitor (**Figure 6C**). And p38 inhibitor SB203580 could recuse cell viability under challenge by TEOA (**Figure S3**). Thus, these results suggest that ROS mediated activation of the p38 MAPK signal pathway plays an important role in initiating TEOA-induced DNA damage.

### TEOA Presented Synergistic Effect With Vindesine or Cyclophosphamide

**Figure 7:**
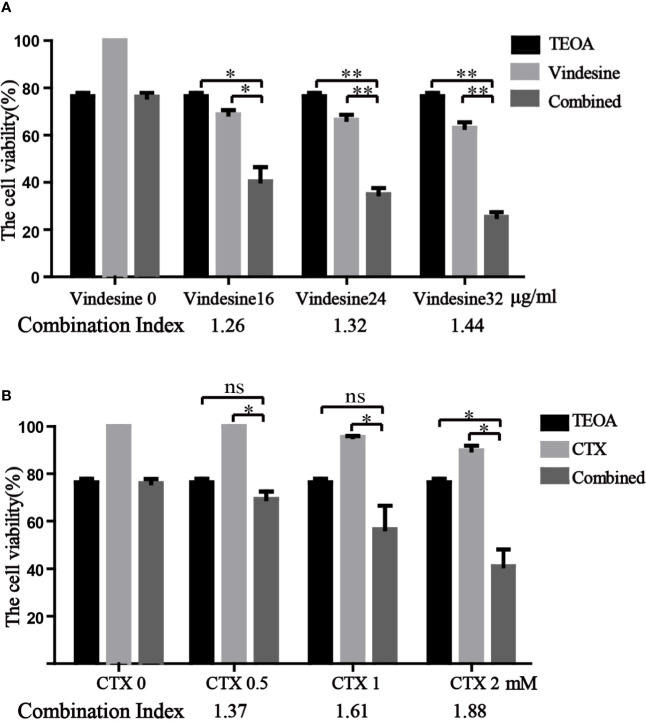
TEOA exhibited a significant synergistic activity with vindesine or cyclophosphamide in diffuse large B-cell lymphoma (DLBCL) cells. **(A, B)** The therapeutic effect of TEOA in combination with vindesine or cyclophosphamide was measured by the CCK-8 assay. OCI-LY10 cells were treated with 15 µM TEOA with or without different concentrations of vindesine or cyclophosphamide for 12 h. And the combination index was calculated below. *P<0.05, **P<0.01, ns= no significance.

Due to the long-term exposure of drugs, drug resistance is inevitable in the treatment of diffuse large B-cell lymphoma (Noble et al., 2017). Important chemotherapeutic drugs of standard chemotherapy regimens, vindesine and cyclophosphamide, were commonly used in patients with DLBCL. Considering the resistance to vindesine and cyclophosphamide, we detected the therapeutic effect of TEOA in combination with vindesine or cyclophosphamide by the CCK-8 assay. Therefore, OCI-LY10 cells were treated with a certain dose of TEOA with or without vindesine or cyclophosphamide. As shown in **Figure 7A**, the viability of OCI-LY10 cells was decreased with TEOA (15 µM) or vindesine (16 µg/ml, 24 µg/ml, 32 µg/ml) treatment alone, but the viability was decreased dramatically when cells were treated with a combination of both. Similar results were also obtained for cyclophosphamide (0.5 mM, 1 mM, 1.5 mM) (**Figure 7B**). In addition, combination index was calculated and revealed that significant synergies between TEOA and chemotherapeutic drugs (Q>1.15) in both cell lines. Further, TEOA exhibits no synergistic effect with vindesine and CTX on immortalized lymphocyte cells (**Figure S4**). and mouse embryonic fibroblast. These results suggest that TEOA may be a promising agent when used in combination with vindesine or cyclophosphamide for the treatment of DLBCL.

## Discussion

In recent years, the morbidity of DLBCL increased significantly in China (Yuan et al., 2014). Chemotherapy is the main treatment used for DLBCL, but long-term chemotherapy causes severe side effects, drug resistance, and a high incidence of disease relapse (Shiozawa et al., 2013). Thus, it is significant to examine potential new drugs for the treatment of DLBCL. The natural medicine showed anticancer effect on most types of cancers (Mei et al., 2017). Moreover, agents isolated from natural sources may have reduced toxicity in the human body. Therefore, it is essential to explore natural drugs with anti-tumor targets, and their molecular mechanism of signal transduction. For decades, traditional Chinese medicine, which has potential of anticancer and immunomodulatory activities, was used to treat gastrointestinal cancer (Satoh, 2014). TEOA is one of the most abundant compounds isolated from the root of *A. eriantha*.

Several studies revealed that TEOA displayed anticancer activities against various human cancer cells (Xin et al., 2010; Fu et al., 2010), and was regarded as an anticancer candidate drug *in vivo* (Gu et al., 2013). In the present study, we found that TEOA has a great inhibitory effect on the viability of OCI-LY3 and OCI-LY10 cells. A large number of studies have demonstrated that ROS exerts its anti-tumor effect through three major pathways: promoting apoptosis of tumor cells, leading to necrosis of tumor cells, and participating in autophagic cell death (Wu et al., 2017; Liu et al., 2017). In this work, ROS generation and apoptosis were detected by flow cytometry. We found that TEOA increased ROS production and promoted apoptosis in DLBCL cells. In addition, TEOA-induced apoptosis could be suppressed by NAC, a ROS scavenger. These results indicate that ROS plays an important role in TEOA-induced apoptosis, and might initiate apoptosis by inducing the generation of ROS.

DLBCL is a heterogeneous disease characterized by high levels of genomic instability (Barlow et al., 2013), and activation of DNA damage repair pathways, including the activation of nucleotide excision DNA repair (NER) and DNA damage response kinases (Shaheen et al., 2011; Gu et al., 2015). Studies have shown that inhibition of the process of DNA damage repair, such as inhibitors of kinase WEE1, could effectively prevent the progress of DLBCL (Knittel et al., 2018; Jong et al., 2020). Furthermore, it has been demonstrated that NER pathway related proteins were usually overexpressed in CHOP (Cyclophosphamide, Doxorubicin, Vincristine and Prednisone) resistant DLBCL cells. Downregulation of these proteins has the potential of reversing drug resistance and improving the efficacy of treatments in DLBCL (Bret et al., 2013). Therefore, inducing DNA damage could be a promising and effective method for DLBCL treatment. Numerous studies have shown that ROS could cause oxidative damage and lead to DNA damage, including DNA strand breaks, DNA site mutations, DNA double-stranded aberrations, proto-oncogenes, and tumor suppressor gene mutations. To investigate the mechanism underlying TEOA-induced cytotoxicity, we studied the effect of TEOA on DNA damage and found that the expression of p53, p21, p27, and γ-H2AX, biomarkers for DNA double-strand breaks repair, increased, which suggested that TEOA induced DNA damage.

P38 is the most important member of the MAPK family with regard to controlling inflammation. It can be activated by physiological stress, lipopolysaccharide, osmotic stress, and ultraviolet radiation. The p38 pathway has an essential relationship with apoptosis. Studies have revealed that p38 can regulate cell death by upregulating the expression of c-myc, promoting phosphorylation of p53, and activating c-Jun pathways (Li et al., 2015; Liu et al., 2015). ROS are activators of the p38 signaling pathway. A previous study reported that ROS could activate the p38 signaling pathway and participate in apoptosis. Therefore, the present study investigated the role of p38 in TEOA-induced apoptosis. After the treatment of TEOA, the protein level of p-p38 significantly increased, indicating that TEOA could activate the p38 pathway. Furthermore, the phosphorylation of CHK1, CHK2, and γ-H2AX were also increased. To further clarify the relationship between P38 activation and DNA damage, we found that the increased expression of DNA damage-related proteins induced by TEOA was dramatically reversed with the administration of NAC and p38 phosphorylation inhibitor SB203580. The above results show that ROS mediates TEOA-induced DNA damage and p38 activation. Additionally, the problem of clinical drug resistance remains a barrier to effective treatment of DLBCL (Wu et al., 2017). The results of the present study indicate that TEOA in combination with vindesine or cyclophosphamide has a synergistic anti-proliferation activity.

In summary, TEOA induced apoptosis and inhibited the growth of DLBCL cells *via* ROS toxicity, which included the activation of the p38. pathway and induction of DNA damage. Moreover, it was evident that TEOA was able to enhance the cytotoxicity of vindesine or cyclophosphamide in DLBCL cell lines. These findings will provide an experimental basis for the application of TEOA in the treatment of clinical DLBCL. However, more studies are needed to achieve a full understanding of TEOA effects *in vivo*.

## Conclusions

This study demonstrates that TEOA inhibits proliferation and induces apoptosis in diffuse large B-cell lymphoma cells through the induction of ROS accumulation, activation of the p38 MAPK signaling pathway, and DNA damage. Moreover, we found that TEOA has a synergistic anti-proliferation activity with vindesine or cyclophosphamide. These data emphasize the potential of TEOA in the clinical application and chemotherapeutic combination therapies in other hematopoietic tumors. However, the detailed mechanisms of the anticancer effect in DLBCL requires further investigation.

## Data Availability Statement

All datasets presented in this study are included in the article/**Supplementary Material**.

## Author Contributions

JD and XY conceived of the presented idea. XiW and XuW developed the theory and performed the computations. XY, XiW and other authors carried out the experiment. XiW and XuW verified the analytical methods. XY wrote the manuscript with support from JD, YW and XT. All authors discussed the results and contributed to the final manuscript.

## Funding

This research was supported by the National Science and Technology Major Project for New Drug (No. 2017ZX301033), the National Natural Science Foundation of China (No. 81570198), the Co-construction of Provincial and Department Project (No. WKJ-ZJ-1709), the Key projects of Zhejiang Provincial Administration of Traditional Chinese Medicine (No. 2016ZZ007, NO.2017ZA011), the Medical and Health Science and Technology Project of Zhejiang Province (No. 2020ZA098, No. 2017KY209, NO.2016RCA002), the Zhejiang Public Welfare Technology Application Research Project (Grant No. LGF19H080006, LGF20H080005, LY18C090004, 2017C33091), and the Medical and Health Science and Technology Project of Zhejiang Province (No. 2019RC014, 2019RC115, 2018KY003, 2017KY006).

## Conflict of Interest

The authors declare that the research was conducted in the absence of any commercial or financial relationships that could be construed as a potential conflict of interest.

The reviewer QS declared a shared affiliation, with no collaboration, with one of the authors, YL, to the handling editor at the time of review.

## Supplementary Material

Figure S1 TEOA reduced DLBCL cell viability and arrest the cell cycle. **(A)** Mouse embryonic fibroblast and immortalized lymphocyte cells were treated with TEOA at indicated concentrations for 12h, cell viability was detected by CCK8 assays. **(B, C)** The quantification of PI positive cells in fig.1E. **(D)** Flow cytometry was used to detect cell cycle in DLBCL cells exposed to increasing concentration of TEOA. **(E)** Cell migration rate was determined by the transwell assay. Corresponding quantified histograms were shown on the right; **P*<0.05.

Figure S2 TEOA increased the level of 8-OHdG. OCI-LY10 cells were treated with indicated concentration of TEOA for 12 h, and the level of 8-OHdG was determined by ELISA. ***P*<0.01.

Figure S3 Inhibition of p38 activation rescued cell viability under treatment of TEOA. SB203580 (10 μM) was pretreatment for 1 h and cotreatment with TEOA for 12 h, cell viability was determined by CCK8 assay. **P*<0.05.

Figure S4 TEOA exhibited no synergistic activity with vindesine or cyclophosphamide in normal cells. TEOA was cotreatment with vindesine or cyclophosphamide for 12 h and cell viability was measured by the CCK-8 assay.

## Abbreviations

BSA, bovine serum albumin; DCF, 2′,7′-dichlorofluorescein; DCFH-DA, 2’,7’-dichlorodihydrofluorescein diacetate; DLBCL, diffuse large B-cell lymphoma; MAPK, mitogen-activated protein kinase; NAC, N-acetyl cysteine; NHL, non-Hodgkin’s lymphomas; PBS, phosphate buffer saline; PI, propidium iodide; PVDF, polyvinylidene fluoride; ROS, reactive oxygen species; SDS-PAGE, sodium dodecyl sulfate polyacrylamide gel electrophoresis; TBST, tris buffered saline tween; TEOA, 2α, 3α, 24-Thrihydroxyurs-12-en-24-ususulic acid.
