# Corrigendum: TEOA inhibits proliferation and induces DNA damage of diffuse large B-cell lymphoma cells through activation of the ROS-dependent p38 MAPK signaling pathway

**DOI:** 10.3389/fphar.2022.973989

**Published:** 2022-08-12

**Authors:** Xingxing Yu, Xin Wang, Xu Wang, Yi Zhou, Yanchun Li, Aiwei Wang, Tongtong Wang, Yihan An, Weidong Sun, Jing Du, Xiangmin Tong, Ying Wang

**Affiliations:** ^1^ Clinical Research Institute, Zhejiang Provincial People’s Hospital, People’s Hospital of Hangzhou Medical College, Hangzhou, China; ^2^ Department of Hematology, Fuyang Hospital of Anhui Medical University, Fuyang, China; ^3^ School of Laboratory Medicine and Life Science, Wenzhou Medical University, Wenzhou, China; ^4^ Department of Laboratory Medicine, Zhejiang Provincial People’s Hospital, People’s Hospital of Hangzhou Medical College, Hangzhou, China; ^5^ Wangjiangshan Institute, Zhejiang Provincial People’s Hospital, People’s Hospital of Hangzhou Medical College, Hangzhou, China; ^6^ The Second Clinical Medical School of Zhejiang Chinese Medical University, Zhejiang Chinese Medical University, Hangzhou, Zhejiang, China; ^7^ Department of Hematology, The First People’s Hospital of Fuyang, Hangzhou, China; ^8^ Phase I Clinical Research Center, Zhejiang Provincial People’s Hospital, People’s Hospital of Hangzhou Medical College, Hangzhou, China

**Keywords:** TEOA, diffuse large B-cell lymphoma, DNA damage, reactive oxygen species, p38 MAPK

In the published article, there was an error in Figures 3E, 4E as published. The images of Vehicle in Figure 3E and TEOA + GSH in Figure 4E were incorrect. The corrected Figures 3E, 4E and its caption appear below.

**FIGURE 3:**
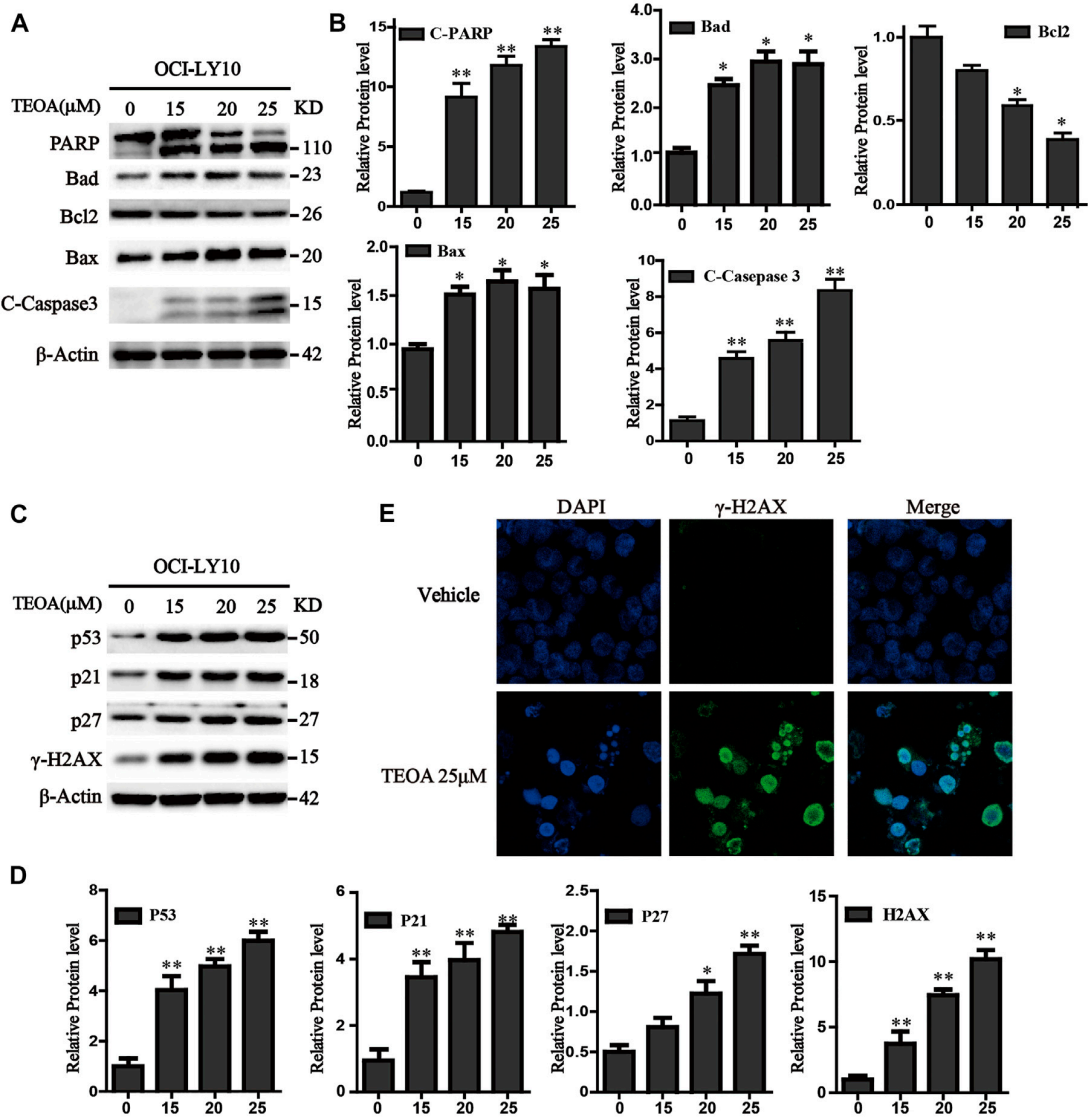
TEOA affected the expression of apoptosis-related protein in OCI-LY10 cells. **(A)** Diffuse large B-cell lymphoma (DLBCL) cells were exposed to various concentrations of TEOA for 12 h. The expression of the following apoptosis-related proteins was determined by western blot: cleaved PARP, caspase-3, and Bcl-2 family members Bcl-2, Bad, and Bax; β-actin was used as a loading control. **(B)** The quantitation of cleaved PARP, caspase-3, Bcl-2, Bad, and Bax were shown. ∗*p* < 0.05; ∗∗*p* < 0.01. **(C)** OCI-LY10 cells were treated with different doses of TEOA for 12 h. The expression of P53, P21, P27, and g-H2AX protein was determined by western blot; β-actin was used as a loading control. **(D)** The quantitation of P53, P21, P27, and γ-H2AX. Data were presented as mean ± SD. ∗*p* < 0.05; ∗∗*p* < 0.01. **(E)** OCI-LY10 cells were treated with 25 μM TEOA for 12 h and stained with γ-H_2_AX (1:200) antibody. DAPI was used for nucleus staining. Images were acquired with the confocal laser scanning microscopy.

**FIGURE 4:**
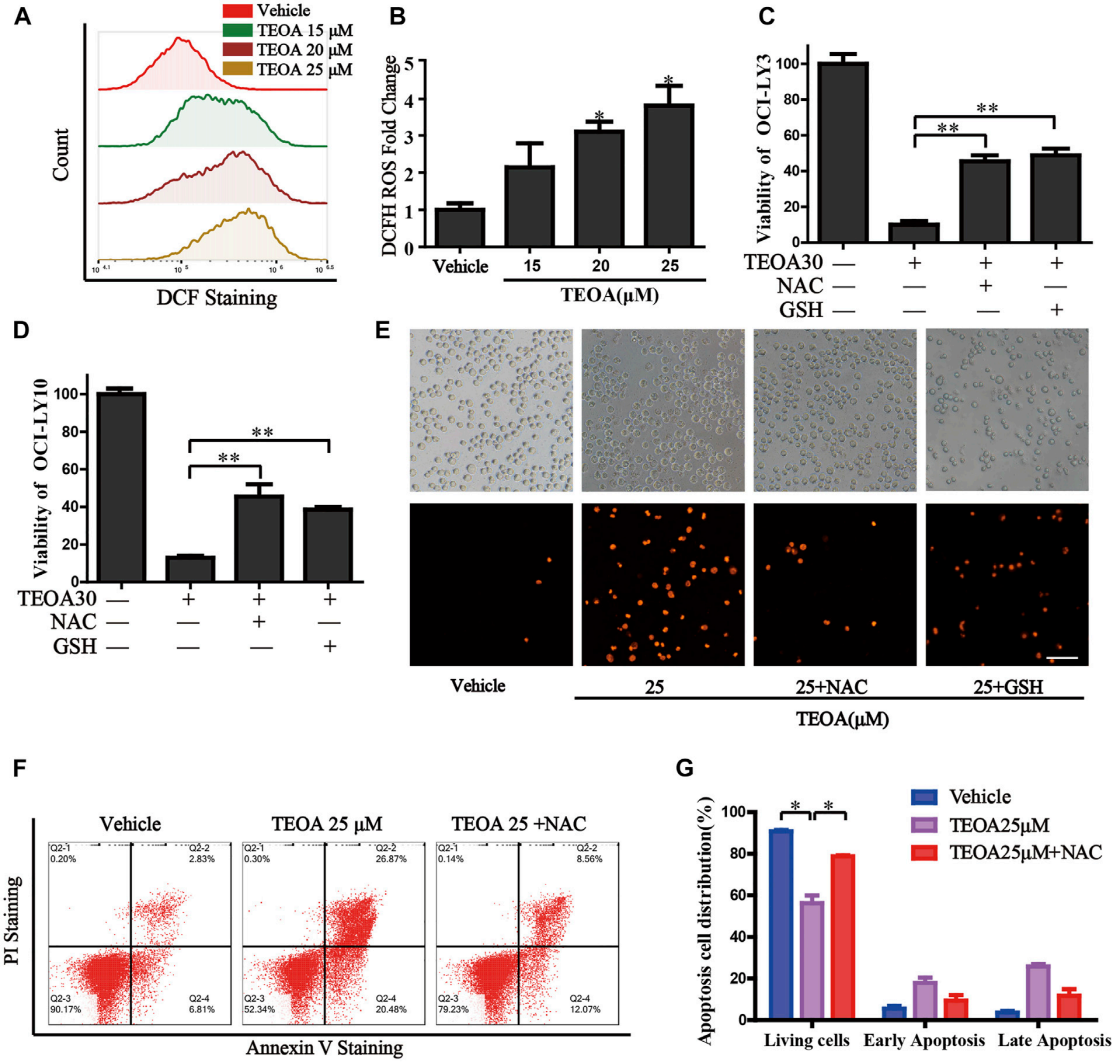
| TEOA increased the production of cellular ROS and promoted apoptosis. **(A)** OCI-LY10 cells were exposed to different concentrations of TEOA for 12 h, then treated cells were stained with DCF-DA for 30 min, cellular ROS levels were determined by flow cytometry. **(B)** Quantitative analysis of the cellular ROS, ∗*p* < 0.05. OCI-LY10 and OCI-LY3 cells were treated with TEOA for 12 h in the presence or absence of NAC and GSH, cell death was determined by CCK8 assay **(C,D)** and PI staining **(E)**. **(F)** Flow cytometry was used to detect apoptosis in DLBCL cells exposed to TEOA with or without NAC treatment. **(G)** The proportion of apoptotic cells was shown on the right, ∗*p* < 0.01.

The authors apologize for this error and state that this does not change the scientific conclusions of the article in any way. The original article has been updated.

