# Supplementary figures and images for: TEOA Inhibits Proliferation and Induces DNA Damage of Diffuse Large B-Cell Lymphoma Cells Through Activation of the ROS-Dependent p38 MAPK Signaling Pathway
Source: Front Pharmacol. 2020 Sep 4;11:554736. doi: 10.3389/fphar.2020.554736 (PMC7500465; doi:10.3389/fphar.2020.554736)

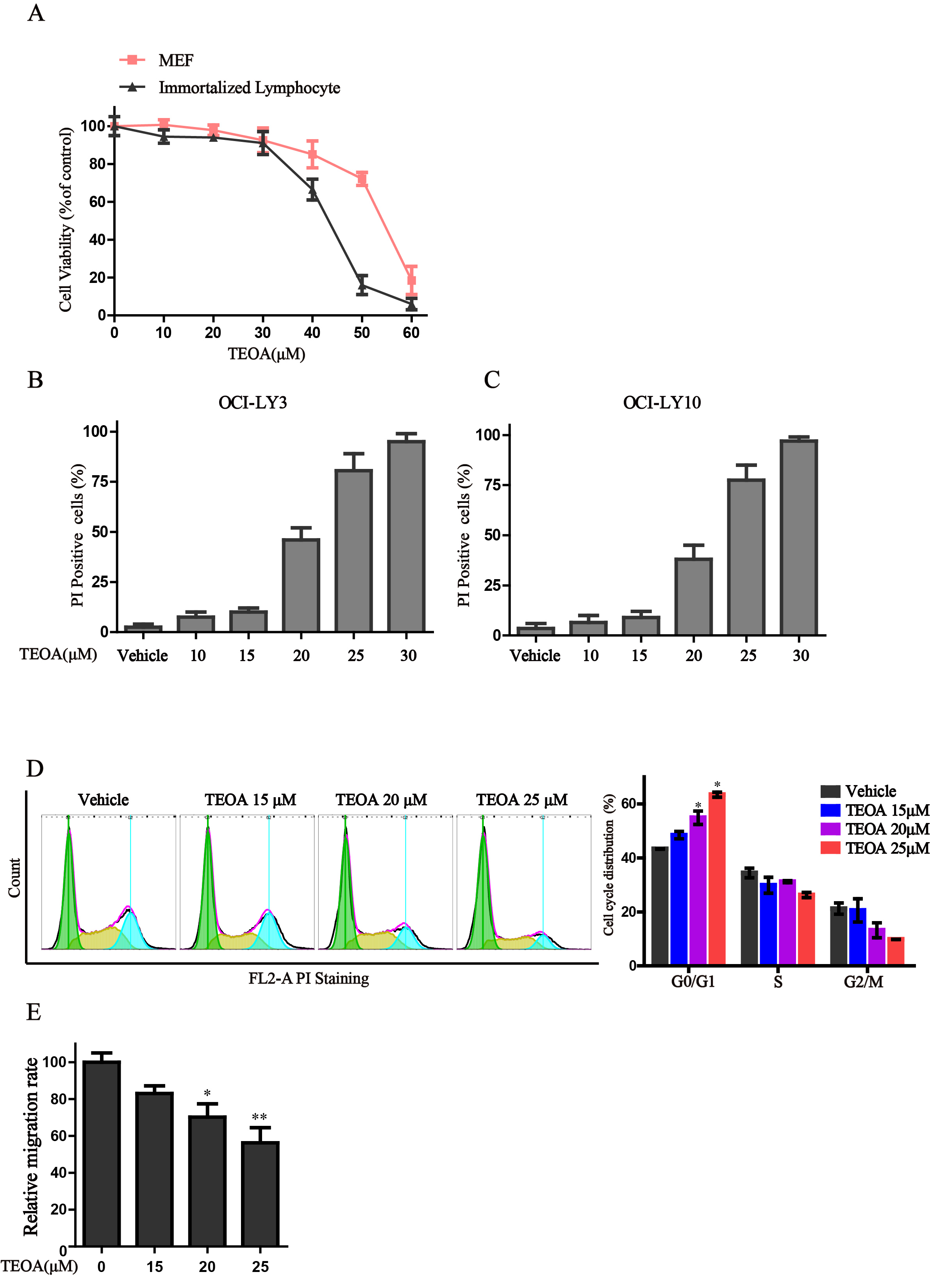

Supplement: Figure S1 — TEOA reduced DLBCL cell viability and arrest the cell cycle. (A) Mouse embryonic fibroblast and immortalized lymphocyte cells were treated with TEOA at indicated concentrations for 12h, cell viability was detected by CCK8 assays. (B, C) The quantification of PI positive cells in fig.1E. (D) Flow cytometry was used to detect cell cycle in DLBCL cells exposed to increasing concentration of TEOA. (E) Cell migration rate was determined by the transwell assay. Corresponding quantified histograms were shown on the right; *P<0.05. [file Image_1.jpeg]

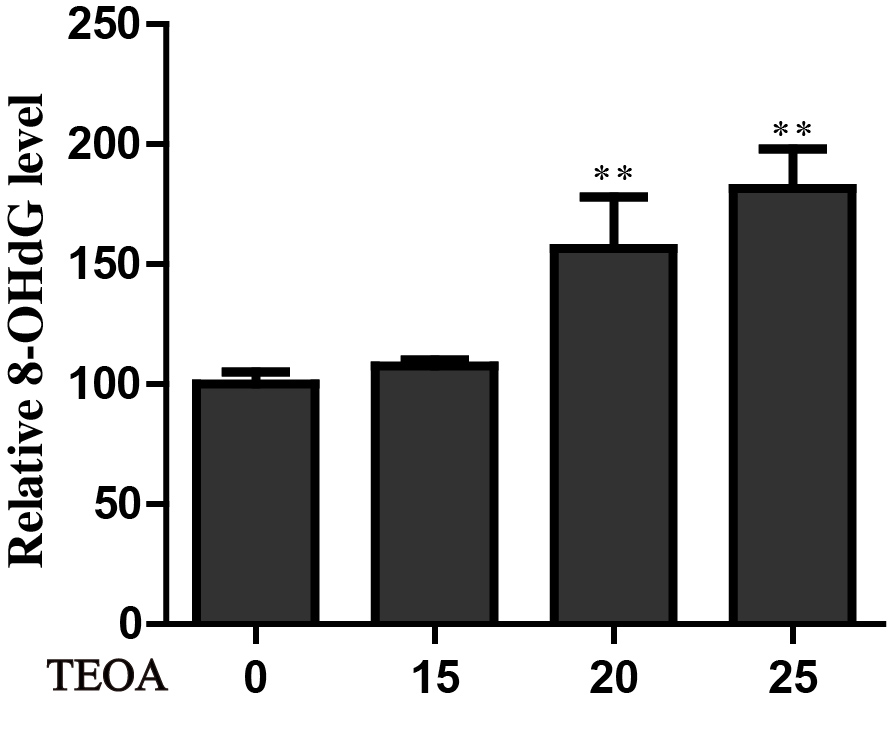

Supplement: Figure S2 — TEOA increased the level of 8-OHdG. OCI-LY10 cells were treated with indicated concentration of TEOA for 12 h, and the level of 8-OHdG was determined by ELISA. **P<0.01. [file Image_2.jpeg]

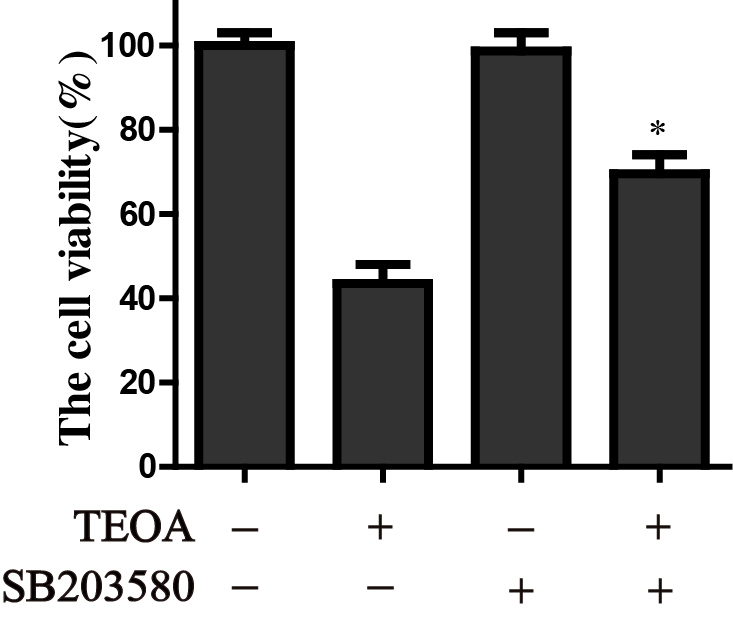

Supplement: Figure S3 — Inhibition of p38 activation rescued cell viability under treatment of TEOA. SB203580 (10 μM) was pretreatment for 1 h and cotreatment with TEOA for 12 h, cell viability was determined by CCK8 assay. *P<0.05. [file Image_3.jpeg]

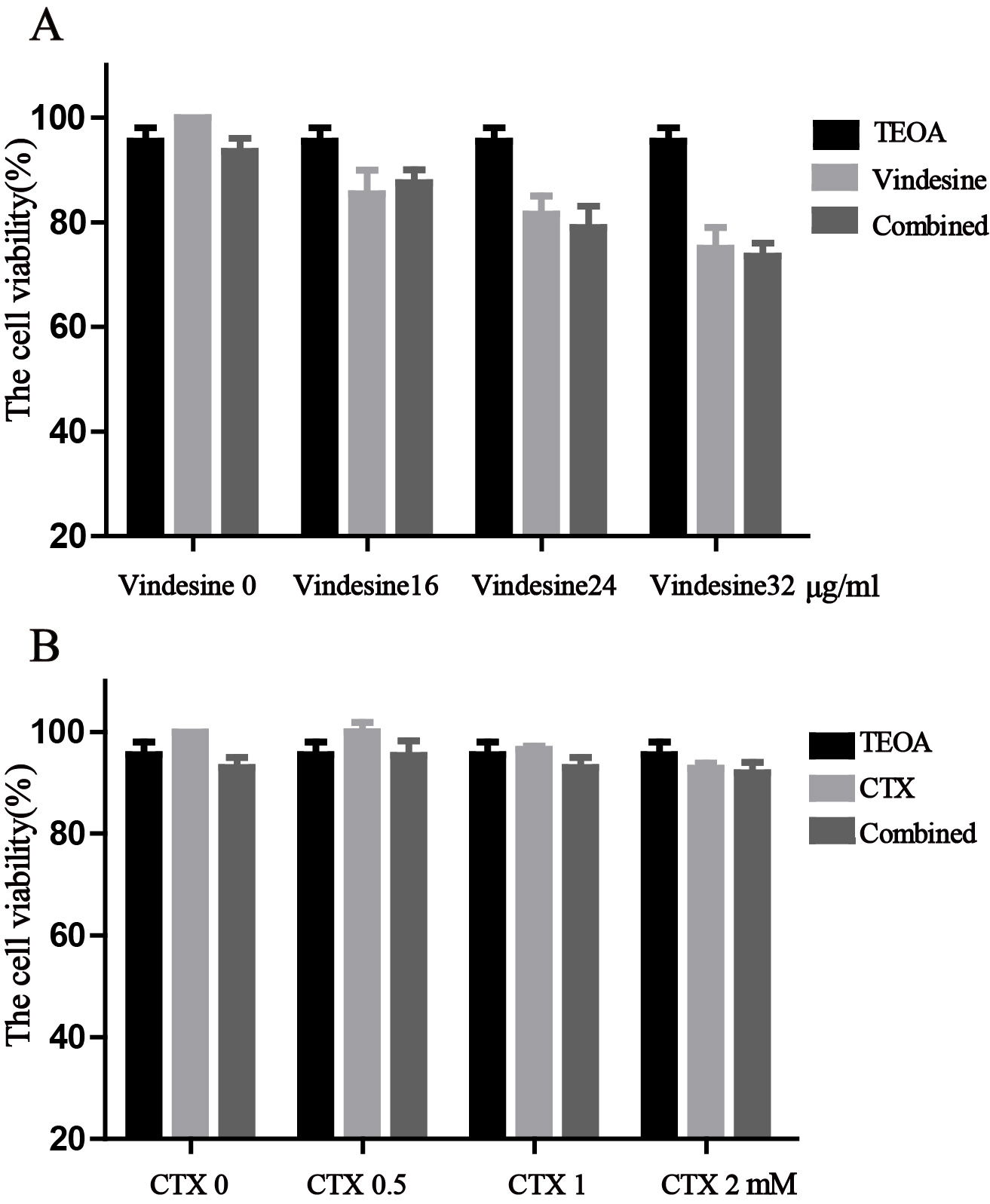

Supplement: Figure S4 — TEOA exhibited no synergistic activity with vindesine or cyclophosphamide in normal cells. TEOA was cotreatment with vindesine or cyclophosphamide for 12 h and cell viability was measured by the CCK-8 assay. [file Image_4.jpeg]
